# Bacterial Outer Membrane Vesicles Induce Vitronectin Release Into the Bronchoalveolar Space Conferring Protection From Complement-Mediated Killing

**DOI:** 10.3389/fmicb.2018.01559

**Published:** 2018-07-13

**Authors:** Magnus Paulsson, Karlhans F. Che, Jonas Ahl, Johan Tham, Linda Sandblad, Margaretha E. Smith, Ingemar Qvarfordt, Yu-Ching Su, Anders Lindén, Kristian Riesbeck

**Affiliations:** ^1^Clinical Microbiology, Department of Translational Medicine, Faculty of Medicine, Lund University, Lund, Sweden; ^2^Unit for Lung and Airway Research, Institute of Environmental Medicine, Karolinska Institutet, Stockholm, Sweden; ^3^Infectious Diseases Research Unit, Department of Translational Medicine, Lund University, Lund, Sweden; ^4^Department of Molecular Biology, Umeå University, Umeå, Sweden; ^5^Institute of Medicine, Sahlgrenska Academy, University of Gothenburg, Gothenburg, Sweden; ^6^Department of Respiratory Medicine and Allergy, New Karolinska Solna, Karolinska University Hospital, Stockholm, Sweden

**Keywords:** alveolar epithelial cells, complement regulators, endotoxin, outer membrane vesicles, *Haemophilus influenzae*, immune evasion, pneumonia, *Pseudomonas aeruginosa*

## Abstract

Pathogens causing pneumonia utilize the complement regulator vitronectin to evade complement-mediated killing. Although vitronectin is associated with several chronic lung diseases, the role of bronchoalveolar vitronectin in pneumonia has not been studied. This study sought to reveal the involvement of vitronectin in the bronchoalveolar space during pneumonia, to assess the effect of outer membrane vesicles and endotoxin on vitronectin release, and to determine whether bacterial pathogens utilize pulmonary vitronectin for evasion. Vitronectin was analyzed in cell-free bronchoalveolar lavage fluid harvested from patients with pneumonia (*n* = 8) and from healthy volunteers after subsegmental endotoxin instillation (*n* = 13). Vitronectin binding by *Pseudomonas aeruginosa* and *Haemophilus influenzae* was analyzed, and subsequent complement evasion was assessed by serum challenge. The effects of outer membrane vesicles on vitronectin production in mouse lungs and human type II alveolar epithelial cells (A549) were determined. We detected increased vitronectin concentrations in lavage fluid during pneumonia (*p =* 0.0063) and after bronchial endotoxin challenge (*p =* 0.016). The capture of vitronectin by bacteria significantly reduced complement-mediated lysis. Following challenge with vesicles, vitronectin was detected in mouse bronchoalveolar space, and mouse alveolar epithelial cells *in vivo* as well as A549 cells *in vitro* contained increased levels of vitronectin. Taken together, outer membrane vesicles and endotoxin from Gram-negative bacteria induce vitronectin, which is released into the bronchoalveolar space, and used for evasion of complement-mediated clearance.

## Introduction

The complex interplay between bacteria and the pulmonary defenses of the host determines the success of the invading pathogens. The respiratory pathogens *Haemophilus influenzae* and *Pseudomonas aeruginosa* cause pneumonia by overcoming the innate immunity of the host through an array of virulence factors. Innate immunity in the lungs includes physical barriers such as mucociliary movement and the epithelial cell lining, antimicrobial peptides, complement proteins, and pathogen-responsive cells (Marc et al., 2004; Bolger et al., 2007; Mizgerd, 2012). A critical part of the host defense in the lungs is the response to pathogen-associated molecular patterns (PAMPs), such as bacterial cell wall components including lipopolysaccharide, that is, endotoxin. PAMPs initiate inflammation, cell recruitment, and clearance of bacteria via lysis or phagocytosis (Parker and Prince, 2011). Nonetheless, pathogens have evolved to evade host defenses through strategies that include enhanced adhesion to the airway epithelium and recruitment of complement-regulatory proteins to decrease the bactericidal effect of serum (Lambris et al., 2008; Singh et al., 2010). Recent years it has been shown that a large part of the pro-inflammatory response induced by PAMPs in the lung is related to outer membrane vesicles. These nanoparticles are released into the bronchial lumen in large amounts, and contain major virulence factors mediating several functions at a distance from the parent bacterium (Sharpe et al., 2011; Park et al., 2013).

Invasive and mucosal bacterial pathogens escape complement-mediated killing by recruiting complement regulators to their cellular surfaces (Su and Riesbeck, 2017). Vitronectin, a 75-kDa glycoprotein found in plasma and the extracellular matrix, is among such complement regulators. Although primarily produced by hepatocytes and released into the circulation, vitronectin can also be generated by other cell types including respiratory epithelial cells (Boyd et al., 1993; Salazar-Peláez et al., 2015; Uhlén et al., 2015). It protects human cells and tissues from self-damage by inhibiting formation of the membrane attack complex (Sheehan et al., 1995). Other functions of vitronectin are associated with cellular attachment and migration, tissue healing, and regulation of apoptosis (Preissner, 1991; Wheaton et al., 2016). Vitronectin is also an effector associated with inflammatory processes, as evidenced by increased levels of the glycoprotein in the bronchial lumen of patients with chronic lung disease (Eklund et al., 1992; Pohl et al., 1993a; Teschler et al., 1993; Carpagnano et al., 2003).

Vitronectin binding surface proteins on respiratory pathogens include nontypeable *H. influenzae* (NTHi) protein E, *P. aeruginosa* OprD, and *Streptococcus pneumoniae* PspC (Hallström et al., 2009; Singh et al., 2010; Voss et al., 2013; Paulsson et al., 2015). By recruiting vitronectin to these surface proteins, microbes inhibit insertion of the membrane attack complex and gain resistance to complement (Bolger et al., 2007; Singh et al., 2010; Voss et al., 2013). Moreover, surface-bound vitronectin can enhance bacterial adherence to the epithelium by facilitating bacteria-host cell-cell interactions (Bergmann et al., 2009; Singh et al., 2014). Vitronectin-dependent virulence in the lungs is underscored by enhanced vitronectin-binding capacity of *P. aeruginosa* isolates from the bronchoalveolar space relative to that of isolates from other infection sites (Paulsson et al., 2015).

Despite the role of vitronectin in the inhibition of complement-mediated killing, regulation of vitronectin release during pneumonia by factors such as bacterial PAMPs remains unknown. The aim of this study was to determine whether vitronectin levels are elevated in the lung during pneumonia, in response to bacterial outer membrane vesicles and endotoxins, and whether pulmonary vitronectin is utilized by the respiratory pathogens to increase fitness.

## Materials and Methods

### Bacterial Strains and Culture Conditions

Nontypeable *H. influenzae* (NTHi) 3655 was cultured in either brain heart infusion broth supplemented with 10 μg/ml nicotinamide adenine dinucleotide and 10 μg/ml hemin or on chocolate agar plates (Su et al., 2013). *P. aeruginosa* PAO1 or clinical strain KR796 from the bronchoalveolar space was grown in lysogeny broth or on blood agar plates (Paulsson et al., 2015).

### Isolation of Outer Membrane Vesicles (OMV)

Outer membrane vesicles were isolated according to Rosen et al. (1995) with an additional purification step. Briefly, bacteria were grown overnight, then removed by centrifugation and filtration through 0.45-μm pore filters. Supernatants were concentrated using Vivaflow 200, 100,000 MWCO (Sartorius, Göttingen, Germany), and OMV were separated by ultracentrifugation at 162,000 RCF and repeated centrifugation using sucrose and density gradient mixture to further remove debris (HistoDenz; Sigma, St. Louis, MO, United States). OMV were suspended in 60% HistoDenz, and a 20–50% gradient was added on top using a gradient-maker (S30; GE Healthcare, Amersham, United Kingdom) followed by further centrifugation steps and collection of OMV (visible as a thin ring approximately 1 cm from the surface). After further centrifugation steps and washing in phosphate-buffered saline (PBS), the purity of OMV preparations was verified by transmission electron micrographs (TEM; demonstrated in Supplementary Figure S1). The control of NTHi OMV purity has been published earlier (Deknuydt et al., 2014). The endotoxin content of OMV was analyzed using the Limulus amoebocyte lysate assay (Endosafe MCS Kinetic Reader; Charles River, Wilmington, MA, United States). OMV preparations contained between 10^5^–10^6^ EU LPS/mg of protein in OMV, as measured by Pierce BCA Protein Assay Kit (Thermo Scientific, Waltham, MA, United States). By approximating 1 EU to 0.1 ng, we estimate the endotoxin content to be 0.01–0.1 mg LPS/mg OMV. OMV from *P. aeruginosa* where generally in the lower end of the range and OMV from NTHi in the higher end.

### Clinical Study Subjects

Study subjects (*n* = 8) were recruited from patients admitted with clinical signs of pneumonia (shortness of breath and any symptoms of fever, purulent sputa, or radiographic infiltrate). Demographic and clinical data of all included patients are summarized in **Table 1**. All subjects were scheduled for bronchoscopy at the Department of Infectious Diseases, Skåne University Hospital (Malmö, Sweden) between September 1st, 2015 and April 30th, 2016. Following the identification of the most affected lung segments, bronchoalveolar lavage fluid (BALF) was collected using 3 × 50 ml PBS. In selected cases, protected bronchial brush specimens were obtained. Patient data were collected from the hospital medical records (Melior; Siemens Healthcare, Upplands Väsby, Sweden) and stored in the online database REDCap hosted at Lund University ^1^ (Obeid et al., 2013).

**Table 1:** Demographic and microbiological data of patients.

Patient ID	1	2	3	4	5	6	7	8
*Age (years)*	81	69	63	59	64	82	39	82
*Sex*	Female	Female	Male	Female	Male	Male	Male	Male
*Smoking habits*	No data	No data	Non-smoker	Non-smoker	Previous smoker	Non-smoker	Non-smoker	Non-smoker
**Underlying conditions**		
*Immunodeficiency*	Neutropenic, steroids	Leukemia	No	No	Steroids, Methotrexate	No	Steroids	Steroids
*Comment*	Acute myeloid leukemia	CLL, Lung cancer	Frequent aspirations	Frequent aspirations	Urinary bladder cancer, RA			Recent pulmonary embolism (PE)
**Current disease**								
*Radiographic lung infiltrate*	Yes	Yes	Yes	No	Yes	Yes	No	Yes
*Purulent secretions*	Not known	No	Yes	No	Not known	Yes	Yes	No
*Temp > 38°C within the last 24h*	Yes	Yes	Yes	No	Yes	Yes	Yes	Yes
*Duration of symptoms (days)*	22	14	2	18	7	13	34	7
*Sepsis*	Severe sepsis	No	Severe sepsis	No	Septic shock	Septic shock	Severe sepsis	Septic shock
*Mechanical ventilator*	No	No	No	No	Yes	Yes	Yes	Yes
*FiO2/Oxygen (L/min)*	-/1	-/1	-/4	-/-	40%/-	60%/-	21%/-	35%/-
*Days on ventilator*					2	5	7	5
**Microbiological data**		
*Isolated microbial species*	*Penicillium species*	*Nocardia farcinica*	*Klebsiella pneumoniae*	*Staphylococcus aureus*	*Pneumocystis jiroveci*	*Legionella pneumophila*	*Staph. aureus*	*Serratia marcescens*
		*Enterococcus faecium*	*Pseudomonas aeruginosa*			Influenza A H1N1		
*Ongoing antibiotic treatment*	Meropenem	Sulfamethoxa-trimethoprim	Imipenem	Cloxacillin	Cefotaxime	Levofloxacin	Sulfamethoxa. - trimethoprim	Cefotaxime
	Clindamycin	Amoxicillin-Clavulanic Acid			Erythromycin	Oseltamivir		Vancomycin
	Voriconazole							

*Characteristics of all patients with pneumonia included in this study. No patient had any chronic pulmonary disease or was treated with inhaled steroids.*

### Endotoxin Exposure of Human Healthy Volunteers

Healthy volunteers (*n =* 14) were recruited for bronchoscopy with endotoxin instillation followed by a second bronchoscopy for BALF collection. This medically safe procedure has been previously described in detail (Glader et al., 2010; Smith et al., 2013). The corresponding segmental bronchi in both lungs were instilled with 10 ml PBS, with one side receiving 4 ng/kg *Escherichia coli* 0113:H10 endotoxin (USP, Rockville, MD, United States). We obtained subject case-specific vehicle controls and data to calculate a study-specific normal range [mean ± 2 standard deviations (SD)]. At 12 or 48 h after instillation, BALF was collected using 3 × 50 ml PBS in each segment. One subject was excluded because of adverse effects (vomiting). The remaining subjects (*n* = 13) had very mild systemic and local symptoms during the first 12 h of observation after exposure.

### BALF Processing and Enzyme-Linked Immunosorbent Assay (ELISA)

BALF samples were filtered through a woven mesh filter (Merck, Darmstadt, Germany) and centrifuged at 300 × *g* for 10 min to obtain cell-free samples. Protein quality and semi-quantitative concentrations were evaluated by sodium dodecyl sulfate polyacrylamide gel electrophoresis (SDS-PAGE) followed by Coomassie Blue staining (Supplementary Figure S2). Extracellular vitronectin concentrations were determined using ELISA according to the manufacturer’s instructions (Thermo-Fisher, Frederick, MD, United States) by extrapolating sample concentrations from a standard curve established using vitronectin standards included in the ELISA Kit (Prism, GraphPad Software, La Jolla, CA, United States).

### Bacterial Vitronectin Acquisition and Utilization

Bronchoalveolar lavage fluid samples were heat-inactivated (56°C for 20 min) and subsequently filter-sterilized using a 0.45-μm pore filter. Bacteria, grown as described above, were resuspended in PBS with 2% bovine serum albumin to OD_600_ = 1. Then, 30 μl of bacterial suspension was mixed with 70 μl BALF and incubated for 1 h at 37°C. To investigate vitronectin binding to the bacterial surface, the samples were incubated with rabbit anti-vitronectin polyclonal antibodies (ab20091; Abcam, Cambridge, United Kingdom) at 1:100 for 1 h in 23°C, after washing steps. This was followed by further washing and incubation with secondary antibodies at 1:100 for 0.5 h at 23°C [FITC-conjugated swine anti-rabbit polyclonal antibodies (Dako, Glostrup, Denmark) for NTHi or Alexa Fluor 647 goat anti-rabbit polyclonal antibodies (a21244; Invitrogen, Eugene, OR, United States) for *P. aeruginosa*]. The primary antibodies were omitted in control samples. The samples were kept on ice until fluorescence was detected by flow cytometry using a 488 nm or 633 nm laser (FACSVerse; BD Biosciences, San Jose, CA, United States).

### Serum Bactericidal Assay

Vitronectin complement inhibitory activity was determined using a serum bactericidal assay as previously described (Su et al., 2013). Briefly, vitronectin-depleted BALF was obtained by incubating equal volume of undiluted BALF with sepharose protein A/G resin (GE Healthcare, Chicago, IL, United States) bound to anti-vitronectin antibodies (1:2) and by subsequently collecting the vitronectin-depleted flow-through. Vitronectin depletion in BALF was evaluated by western blotting and ELISA (Supplementary Figure S3). Complement activity in BALF was stopped by heat inactivation (56°C, 30 min). Bacterial cells (1.5 × 10^3^ CFU), from culture grown as described above, were washed by centrifugation (10,000 RCF), resuspended in 20 μl PBS and preincubated for 1 h in 100 μl BALF or vitronectin-depleted BALF. The bacteria were then challenged with normal human serum titrated to 5% for NTHi and to 10% for *P. aeruginosa*. Surviving bacteria were determined by counting colony-forming units.

### Stimulation of Epithelial Cells With OMV and Detection of Surface-Bound Vitronectin

Type II alveolar cells (A549; 5 × 10^4^) were cultured in 24-well plates in F-12 medium (Life Technologies, San Diego, CA, United States) with 10% fetal bovine serum and 5 μg/ml gentamicin at 37°C with 5% CO_2_. Before initiation of experiments, cells were starved in F12 for 16 h. Outer membrane vesicles from NTHi or *P. aeruginosa* in F-12 were added to the cells (titrated 0.5–5 μg/ml). At selected time points, the cells were harvested using Accutase (Innovative Cell Technologies, San Diego, CA, United States) for subsequent flow cytometry. Surface-bound vitronectin on A549 cells was analyzed using the anti-vitronectin mouse monoclonal antibody VN58-1 (Abcam) and FITC-conjugated rabbit anti-mouse polyclonal antibodies (Dako). The specificity of the primary antibody was evaluated by western blotting. Fluorescence was detected by flow cytometry (FACSVerse).

### Isolation of RNA, Reverse Transcription, and Quantitative RT-PCR Analysis

Type II alveolar cells were grown and stimulated with OMV as described above. At selected time points, the cells were lysed using Buffer RLT (Qiagen, Hilden, Germany) for the isolation of total RNA using the RNeasy Mini Kit (Qiagen) according to the manufacturer’s protocol. cDNA was synthesized from 1 μg of total RNA, using the High-Capacity RNA-to-cDNA kit (Life Technologies). *VTN* mRNA levels were quantified using the Prism 7500HT instrument (Applied Biosystems, Foster City, CA, United States), with β-actin used as an endogenous control. The primer sequences for *VTN* were 5′-GGCTGTCCTTGTTCTCCAGTG-3′ (forward) and 5′-GTGCGAAGATTGACTCGGTAGT-3′ (reverse), and sequences for β-actin were 5′-CTGGGACGACATGCAGAAAA-3′ (forward) and 5′-AAGGAAGGCTGGAAGAGTGC-3′ (reverse). The samples were manually loaded into MicroAmp Fast Optical 96-Well Reaction Plates (Applied Biosystems) and run in technical duplicates. The reaction mixtures (10 μl) contained 5 pmol of each forward and reverse primer, Fast SYBR Green^®^ Master Mix (Applied Biosystems), template cDNA (4 ng), and water. Relative expression was calculated using the ΔΔC_t_ method and presented as fold-change, where the values for the vehicle-exposed samples were set to one.

### Challenge of a Rodent Pulmonary Clearance Model With OMV and Immunohistochemistry

Eight week-old BALB/c mice (Janvier, Saint-Berthevin Cedex, France) were challenged with intranasal PBS containing 50 μg of OMV from *P. aeruginosa* PAO1 (*n =* 10) or NTHi 3655 (*n* = 10) or with PBS only (*n* = 15). At 0, 24, or 48 h following the challenge, mice were sacrificed, and lungs were retrieved in formaldehyde and sliced into 4 μm sections. For immunohistochemistry staining and counterstaining with hematoxylin, primary anti-mouse vitronectin antibodies (ab62769; Abcam) and EnVision FLEX reagents in an Autostainer Plus system were used according to the manufacturer’s protocol (Dako). Antigen retrieval was performed in a pressure cooker in the Target Retrieval Solution, Citrate pH 6 (Dako).

### Statistical Analyses

Statistical differences were analyzed using Wilcoxon matched-pairs signed rank test, Mann–Whitney test, ANOVA, or *t*-test, as appropriate (GraphPad Prism). For all experiments, *p*-values ≤ 0.05 were considered significant.

### Ethics Statement

The cohort studies utilized for the current publication were scrutinized and approved by the Regional Committee for Ethical Review in Gothenburg (T683-07) and Lund (2014/529), Sweden. Oral and written informed consent was obtained from all study subjects, in accordance with the Helsinki declaration. The experiments utilizing animals were approved by the Swedish Board of Agriculture (Lund and Malmö Tingsrätt dnr. 4438/2017).

## Results

### Vitronectin Levels Are Elevated in BALF Collected From Clinical Pneumonia Cases

To measure vitronectin in the bronchoalveolar space during pneumonia, BALF was obtained from patients (*n* = 8) with clinical signs of pneumonia at the time of inclusion (Musher and Thorner, 2014). All enrolled patients were treated with antibiotics (**Table 1**). The isolated microbial species suggested diverse etiologic agents and several cases of polymicrobial infections. One patient (#4) had symptoms that were later attributed to aspiration and not to infection, although *Staphylococcus aureus* was cultured from a protected brush specimen.

**FIGURE 1:**
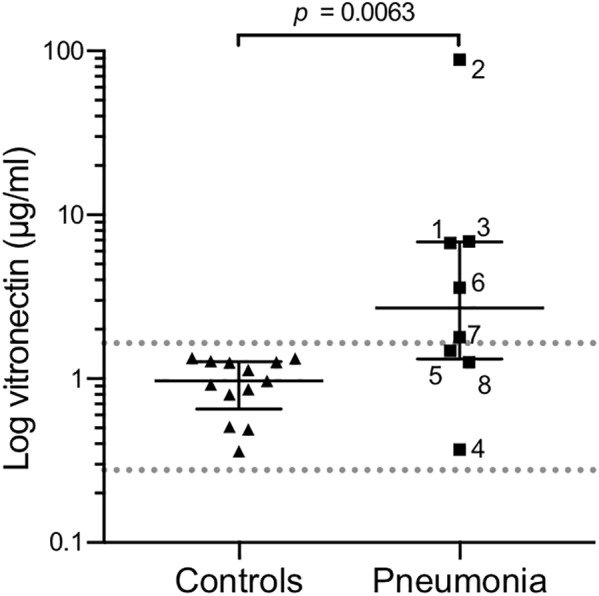
Bronchoalveolar lavage fluid (BALF) samples from patients with pneumonia contain elevated extracellular vitronectin concentrations. BALF samples were collected from patients with pneumonia (*n* = 8) and healthy control subjects (*n* = 13). Vitronectin concentrations were determined by ELISA, with higher values reflecting higher vitronectin concentrations in the bronchoalveolar space. Each point represents one study subject, with triangles representing healthy subjects and squares representing patients with pneumonia. Numbers next to squares refer to the study ID of pneumonia patients. Median and interquartile values are marked with horizontal lines. The dotted lines represent the study-specific reference range (mean ± 2SD) of healthy controls. *p*-values were determined using the Mann–Whitney test.

Patients with pneumonia had markedly increased BALF concentrations of vitronectin (median 2.70 μg/ml, range 0.37–88.7 μg/ml) compared with the 13 healthy control subjects (median 0.97 μg/ml, range 0.36–1.34 μg/ml), as revealed by ELISA (**Figure 1**). The study-specific reference range for vitronectin calculated using the control samples was determined to be 0.28–1.65 μg/ml. Five of the pneumonia patients exhibited vitronectin concentrations exceeding this interval, with patient #2 demonstrating strongly elevated levels (88.7 μg/ml). Taken together, these results indicated that the vitronectin concentrations in BALF samples were significantly higher in patients with clinical pneumonia relative to those in healthy controls.

### Bronchial Endotoxin Challenge Increases Pulmonary Vitronectin Concentrations in Healthy Individuals

**FIGURE 2:**
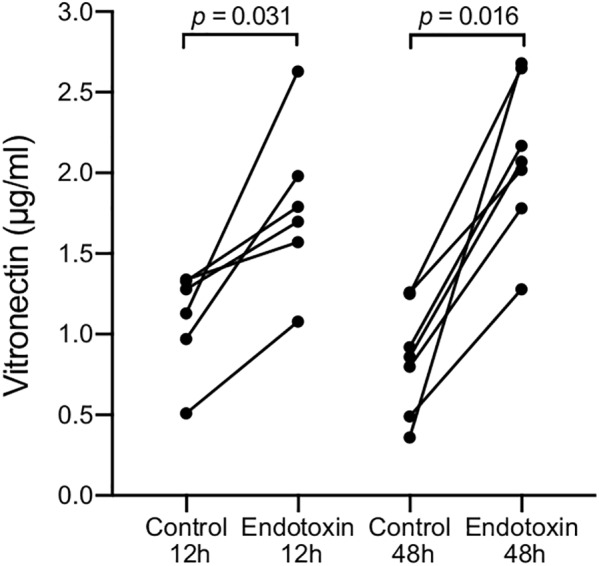
Endotoxin instillation increased extracellular vitronectin concentrations in BALF. Healthy volunteers (*n* = 13) were bronchially exposed to endotoxin in one lung and to PBS in the contralateral lung segment. BALF samples were collected from both exposure sites 12 h (*n* = 6) or 48 h (*n* = 7) after endotoxin challenge. Values from the same individual are connected with a line. Vitronectin concentrations were determined by ELISA, and *p*-values were calculated using the Wilcoxon matched-pairs signed rank test.

To determine if bacterial PAMPs caused the observed elevated vitronectin levels, we recruited healthy volunteers (*n* = 13) for bronchial endotoxin challenge. One lung of each subject was subsegmentally instilled with endotoxin, whereas the contralateral segment received PBS as a case-specific control. BALF was sampled from each segment after 12 h (*n* = 6) or 48 h (*n* = 7). At both sampling times, vitronectin concentrations were significantly increased in all BALF samples from the endotoxin-exposed bronchial segments compared with the corresponding samples from the unexposed contralateral segments (**Figure 2**). Vitronectin concentrations increased 1.5-fold (from median 1.20 μg/ml, range 0.51–1.34 μg/ml to median 1.75 μg/ml, range 1.08–2.63 μg/ml) and 2.4-fold (from median 0.86 μg/ml, range 0.36–1.26 μg/ml to median 2.07 μg/ml, range 1.28–2.68 μg/ml) in endotoxin-exposed segments of individuals sampled 12 and 48 h after the challenge, respectively.

### Bacterial Respiratory Pathogens Utilize Vitronectin From the Bronchoalveolar Space to Acquire Serum Resistance

**FIGURE 3:**
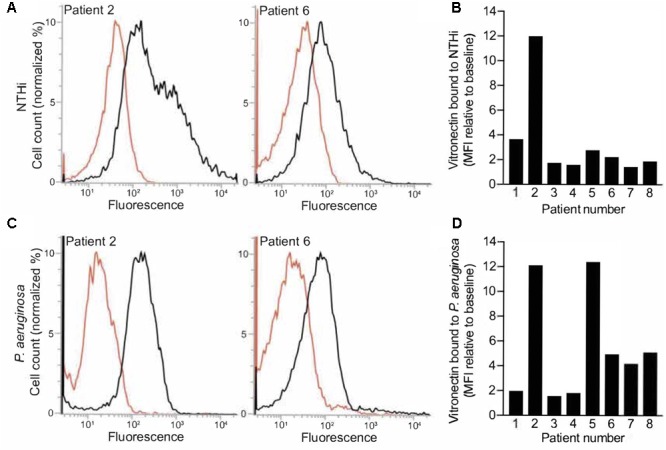
Bacterial respiratory pathogens capture vitronectin from BALF. Binding of vitronectin by nontypeable *H. influenzae* (NTHi) 3655 **(A,B)** and *P. aeruginosa* KR796 **(C,D)** incubated in BALF from pneumonia patients was determined by flow cytometry using primary anti-vitronectin and secondary fluorescent antibodies. Control samples were incubated with secondary antibodies only. **(A,C)** Histograms of two experiments, representing BALF from different patients, for each bacterial species are shown. Control samples are indicated in red. **(B,D)** The change in fluorescence intensity of test relative to control samples reveals that bacteria bound vitronectin in all patient BALF samples. Bars represent mean values of two biological replicates.

We have recently shown that respiratory tract pathogens bind vitronectin to the cell surface *in vitro* (Singh et al., 2014; Hallström et al., 2016). To assess whether vitronectin was available at sufficient levels for bacterial binding in the BALF samples and was not quenched or inhibited by other components, we incubated NTHi 3655 and *P. aeruginosa* KR796 with BALF obtained from pneumonia patients. Cell-bound vitronectin was detected by flow cytometry using primary anti-vitronectin and fluorescent secondary antibodies, with only the secondary antibodies used in the control samples (**Figure 3**). Both NTHi and *P. aeruginosa* bound vitronectin in the BALF samples, as revealed by mean fluorescence intensity increases relative to the control samples. The levels of bacterial vitronectin binding were generally proportional to BALF levels of vitronectin measured by ELISA.

**FIGURE 4:**
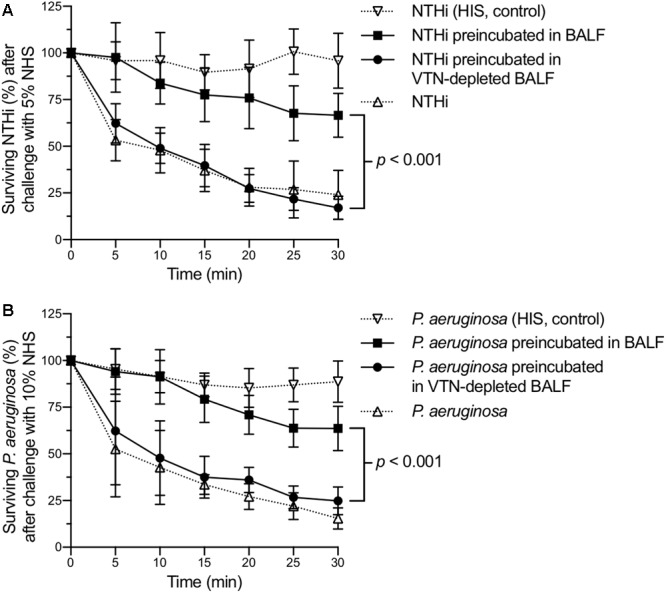
Bacterial respiratory pathogens use vitronectin to evade killing by complement. Nontypeable *H. influenzae* **(A)** and *P. aeruginosa* **(B)** were challenged with normal human serum after preincubation in BALF from pneumonia patients (solid squares) or in vitronectin (VTN)-depleted BALF (solid circles). Bacteria incubated with heat-inactivated serum (HIS) (inverted open triangles) and untreated bacteria (open triangles) were used as controls. Mean values and standard deviations of three biological replicates are shown. Variations between the preincubated groups were analyzed by two-way ANOVA and are presented as *p*-values.

Bacterial pathogens dwelling in the respiratory tract utilize cell-bound vitronectin to inhibit the complement-mediated terminal pathway and to evade killing by innate immunity (Singh et al., 2010). Thus, we examined the biological activity of the vitronectin captured from the BALF samples in a serum bactericidal assay. NTHi or *P. aeruginosa* cells were preincubated in BALF or vitronectin-depleted BALF. Preincubation of bacteria with vitronectin-containing BALF significantly increased survival following exposure to normal human serum relative to preincubation with vitronectin-depleted BALF (**Figures 4A,B**). Upon exposure to human serum for 30 min, 3.9-fold and 2.6-fold more NTHi and *P. aeruginosa* cells, respectively, survived when preincubated with vitronectin-containing BALF than when preincubated with vitronectin-depleted BALF. These experiments confirmed that the bacterial cells captured biologically active vitronectin from BALF to escape complement-mediated lysis.

### Outer Membrane Vesicles Derived From *H. influenzae* or *P. aeruginosa* Induce Vitronectin Release in the Rodent Bronchoalveolar Space

Our results indicated that endotoxin challenge induces vitronectin release in the lungs of healthy individuals. However, endotoxins trigger an immune response in the airway epithelium specifically via Toll-like receptor 4 (TLR4). In contrast, OMV, or bacteria-derived nano-sized spherical structures composed of a phospholipid membrane with surface-exposed membrane-bound proteins and endotoxins, are recognized by several pattern recognition receptors on mammalian cells (Alaniz et al., 2007; Sharpe et al., 2011). Considering that OMV contain bacterial PAMPs other than endotoxins, including outer membrane proteins and peptidoglycans, they more closely reflect the composition of the Gram-negative bacterial cell surface than endotoxins alone.

**FIGURE 5:**
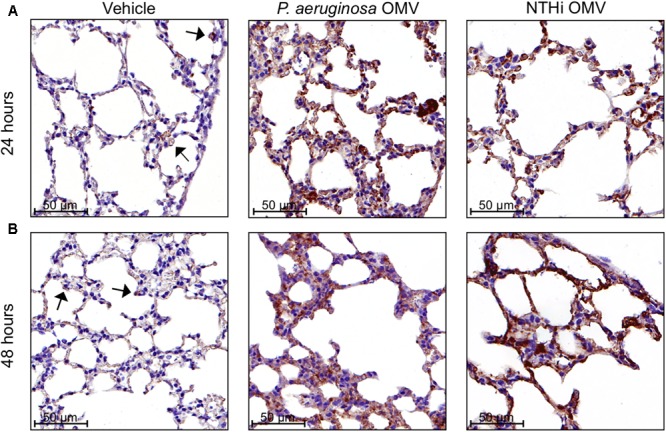
Bacterial outer membrane vesicles (OMV) trigger higher concentrations of vitronectin in the rodent bronchoalveolar space. BALB/c mice were challenged with 50 μg of OMV from nontypeable *H. influenzae* or *P. aeruginosa* or with a vehicle control as indicated, and sacrificed 24 h **(A)** or 48 h **(B)** after the challenge. The pulmonary tissues were stained using immunohistochemistry. Vitronectin was stained brown. Brown staining of type II alveolar cells in the vehicle control images suggested low-level baseline vitronectin production in the alveolar epithelium (indicated with arrows). OMV challenged tissues contained more vitronectin than controls. Images are representative of three biological replicates.

Thus, to further study the appearance of vitronectin in the lungs following bacterial stimuli, BALB/c mice where challenged with OMV isolated from *H. influenzae* and *P. aeruginosa.* Other cellular components, such as flagella, were removed during the preparation of these vesicles (Supplementary Figure S1). Immunohistochemistry was used to analyze vitronectin levels in the lung tissues of BALB/c mice harvested 0, 24, and 48 h after OMV challenge. Following 24 h, OMV from either species caused an increase in immunoreactivity for vitronectin in the alveolar epithelium and in the interstitial space. In contrast, no increase in immunoreactivity was observed in lung tissues from control mice receiving PBS (**Figure 5A**). Following 48 h after OMV exposure, we observed signs of inflammation including thickened alveolar walls due to plasma exudation bringing plasma components including vitronectin to the tissues (**Figure 5B**). Vitronectin was also present in the proximal bronchial lumen, but no intracellular vitronectin was detected inside bronchial epithelial cells. It was, however, present on the ciliated apical surface of both OMV-challenged rodent bronchial epithelial cells and of human bronchial epithelial cells collected from patients with pneumonia (Supplementary Figures S4, S5). In contrast, type II alveolar cells contained vitronectin already at time 0, suggesting that these cells produce vitronectin locally in the bronchoalveolar space.

### Type II Alveolar Cells Produce Vitronectin *in Vitro* Upon Stimulation by OMV

**FIGURE 6:**
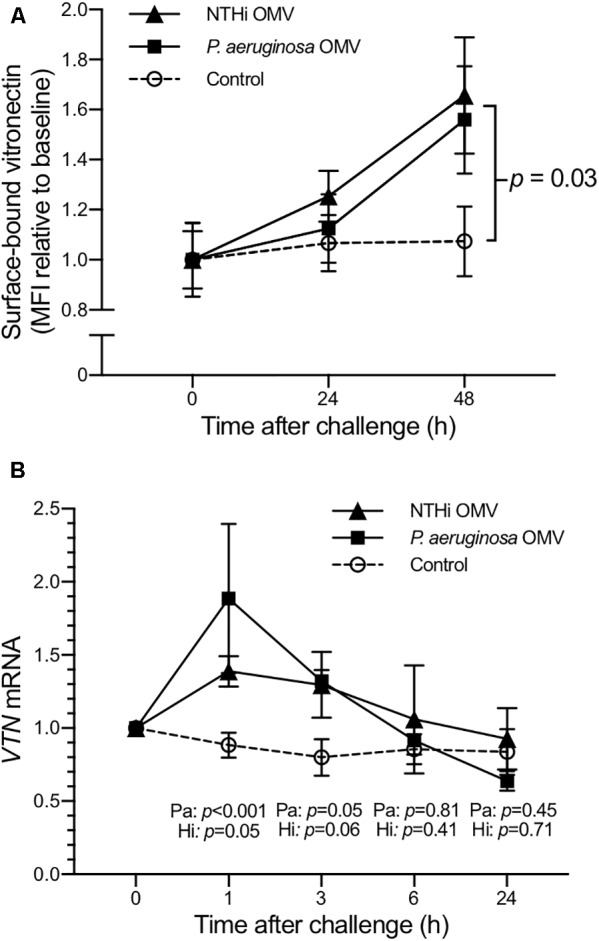
Human type II alveolar cells (A549) produce vitronectin after stimulation with bacterial OMV. Surface-bound vitronectin was detected on A549 epithelial cells by flow cytometry **(A)**. Cells were starved for 18 h prior to the experiment. Following the addition of OMV (5 μg) from *P. aeruginosa* (solid squares) or nontypeable *H. influenzae* (solid triangles), the cells were harvested at indicated time points. Untreated cells were used as controls (open circles). Mean values of two biological replicates are shown, each comprising three technical repetitions. ANOVA was used to calculate the *p*-values. A corresponding increase in vitronectin (*VTN*) mRNA levels was seen 1–3 h after challenge with 1 μg OMV from either bacterial species **(B)**. Symbols represent mean values and error bars represent standard error of the mean of three biological replicates. The difference in mean values between respective bacterial species and controls were assessed using a *t*-test.

To confirm that human type II alveolar cells have the capacity to produce vitronectin, A549 type II alveolar cells were challenged with purified OMV. As revealed by flow cytometry, OMV-challenged A549 cells demonstrated increased surface-bound vitronectin levels relative to control cells (**Figure 6A**). The OMV-challenged A549 cells also exhibited an increase in vitronectin (*VTN*) mRNA levels (**Figure 6B**). Increased levels of *VTN* mRNA were observed as early as 1 h following bacterial OMV-dependent stimulation. After an initial peak at 1 h post-challenge, *VTN* mRNA levels began to decrease at 3 h and returned to baseline by 6 h. Taken together, these experiments confirmed that type II alveolar cells also constitute a pulmonary source of vitronectin, the production of which is triggered by bacterial components present in OMV, albeit the major increase of vitronectin in the lung most likely is related to plasma exudation secondary to inflammation.

## Discussion

We present evidence that vitronectin is an “acute phase” protein in the lungs of humans and mice, the levels of which are elevated in the human bronchoalveolar space during clinical pneumonia. We also show that vitronectin production/plasma exudation can be triggered by *H. influenzae* and *P. aeruginosa* OMV in mice lungs and in human cell cultures, and by purified endotoxins in human lungs. Increased vitronectin was found to be beneficial for the respiratory pathogens studied, as bacterial cells utilized vitronectin released in the bronchoalveolar space to increase resistance against the bacteriolytic complement proteins in human serum.

The concentrations of vitronectin were elevated in BALF samples isolated from patients with pneumonia. Similar levels could be induced in healthy individuals via subsegmental bronchial instillation of endotoxin (**Figures 1, 2**). Patients with polymicrobial infections exhibited some of the highest vitronectin levels. Overall, the heterogeneous microbiological data suggested that increase in vitronectin levels is a global response to invading microbes. The identification of the indicated etiologic microorganisms, based on cultures from protected brush specimens, was not fully complete. For instance, molecular microbiology or antigen tests for *S. pneumoniae* were not included. Two outliers were identified among the patients with signs of suspected pneumonia. The patient with the lowest measured vitronectin concentration (#4) indicated no smoking history, did not suffer from any chronic pulmonary disease, but had frequent aspirations after a severe cerebrovascular insult. *S. aureus* was cultured from a protected brush specimen 18 days prior to testing. At that time, the patient was treated with cloxacillin, despite lack of purulent sputa, radiographic pulmonary infiltrates, or fever. The second outlier, patient #2, demonstrated a dramatically increased concentration of vitronectin in the BALF sample. This patient indicated a previous smoking history and has been treated for lung carcinoma. At the time of inclusion, the patient had pulmonary nocardiosis and local colonization with *Enterococcus faecium*. However, death of the patient shortly after inclusion prevented the determination of the condition responsible for the remarkably high vitronectin concentration in the BALF sample.

All healthy volunteers subjected to endotoxin instillation displayed increased concentrations of vitronectin in BALF samples harvested 12 h after exposure. The elevated vitronectin concentrations, persisting 48 h after exposure, were limited to the lung that was exposed to the endotoxin (**Figure 2**). This experimental setup mimicked the presence of Gram-negative bacteria in the respiratory tract, where endotoxins are present on bacterial cell surfaces and OMV. However, endotoxin exposure allows for the analysis of only TLR4-dependent responses. OMV were hence used to simulate bacterial exposure using mouse and *in vitro* experiments (**Figures 5, 6**). The findings from the mouse experiments, suggesting that type II alveolar cells are a source of vitronectin, were confirmed *in vitro* using the human cell line A549. These alveolar epithelial cells are likely responsible for the early increase in vitronectin levels in the bronchoalveolar space upon bacterial stimulation. The later phase of vitronectin induction may be dependent on extravasation of plasma components, as evidenced by the influx of neutrophils and the thickening of the alveolar interstitium.

Differences in sampling and analysis conditions between previous studies of pulmonary vitronectin prevent meaningful comparisons of observed concentrations. In the present study, the sampling of BALF was standardized to three aliquots of 50 ml PBS to enable comparison between study cases and controls. The average pulmonary vitronectin levels presently detected in patients with pneumonia were similar to those reported for subjects with untreated sarcoidosis (Pohl et al., 1993b).

Our results suggest that *P. aeruginosa* and *H. influenzae* bind vitronectin in the bronchoalveolar space and use it to evade complement-mediated killing (**Figures 3, 4**). BALF samples from pneumonia patients conferred complement resistance to the bacterial cells, whereas vitronectin-depleted BALF samples from the same patients did not. The vitronectin concentration was decreased by approximately 70%. The residual amount of vitronectin left in the vitronectin-depeleted BALF was not sufficient to effectively protect the bacteria against the complement-mediated attack. Partial reduction of other complement regulators (e.g., factor H or C4BP) during vitronectin depletion cannot be excluded. This seems however unlikely considering that the general protein composition was unchanged after vitronectin depletion (Supplement Figure S3). These findings suggest that vitronectin plays a major role in the evasion of the pulmonary host defense.

In addition to complement-regulatory properties, previous studies have reported antimicrobial activity of vitronectin-derived fragments, but not of the surface-bound full-length protein (Malmsten et al., 2006; Singh et al., 2010). We found that the capture of vitronectin from BALF samples *ex vivo* was protective for Gram-negative bacteria. Despite PBS-dilution and the potential presence of interfering molecules, the BALF samples contained vitronectin at concentrations sufficient to promote bacterial capture and to facilitate complement evasion.

## Conclusion

We present several lines of original evidence that vitronectin levels are increased in the bronchoalveolar space during pneumonia due to bacterial surface components including endotoxins that is a major component also in OMV. As indicated in our study, in addition to plasma exudation, vitronectin is also produced *de novo* by alveolar epithelial cells upon stimulation by bacterial OMV. The respiratory bacterial pathogens *H. influenzae* and *P. aeruginosa* subsequently capture and use the available vitronectin to evade serum bactericidal activity and to persist in the mammalian host. This study thus unravels an intricate host-pathogen interaction where the innate immunity system senses the presence of bacteria and their OMV, responds to it, and provides the bacteria with instruments to evade the antimicrobial effects of the complement system.

## Author Contributions

MP, AL, and KR planned the study. MP, KC, LS, MS, IQ, and Y-CS contributed to the experimental work. JA and JT took care of pneumonia patients and did the BALF collection. MP, KC, AL, and KR wrote the manuscript.

## Conflict of Interest Statement

The authors declare that the research was conducted in the absence of any commercial or financial relationships that could be construed as a potential conflict of interest.

## Footnotes

1 www.project-redcap.org
