# Supplementary material for: Bacterial Outer Membrane Vesicles Induce Vitronectin Release Into the Bronchoalveolar Space Conferring Protection From Complement-Mediated Killing
Source: Front Microbiol. 2018 Jul 13;9:1559. doi: 10.3389/fmicb.2018.01559 (PMC6055051; doi:10.3389/fmicb.2018.01559)
Supplement: Supplementary file 1 [file Data_Sheet_1.pdf]

Supplementary data

Bacterial outer membrane vesicles induce vitronectin release into the bronchoalveolar space conferring protection from complement-mediated killing

Magnus Paulsson[1], Karlhans Che[2], Jonas Ahl[3], Johan Tham[3], Linda Sandblad[4], Margaretha E Smith[5], Ingemar Qvarfordt[5], Yu-Ching Su[1], Anders Lindén[2,6], and Kristian Riesbeck[1]

[1]Clinical Microbiology, Department of Translational Medicine, Faculty of Medicine, Lund University, Sweden; [2]Unit for Lung and Airway Research, Institute of Environmental Medicine, Karolinska Institute, Stockholm, Sweden; [3]Infectious Diseases Research Unit, Department of Translational Medicine, Lund University, Sweden; [4]Department of Molecular Biology, Umeå University, Umeå, Sweden; [5]Institute of Medicine, Sahlgrenska Academy at the University of Gothenburg, Gothenburg, Sweden; [6]Lung Allergy Clinic, Karolinska University Hospital, New Karolinska Solna, Stockholm, Sweden

Running title: Bacterial outer membrane vesicles trigger vitronectin release into the bronchoalveolar space

Correspondence

Kristian Riesbeck:

kristian.riesbeck@med.lu.se

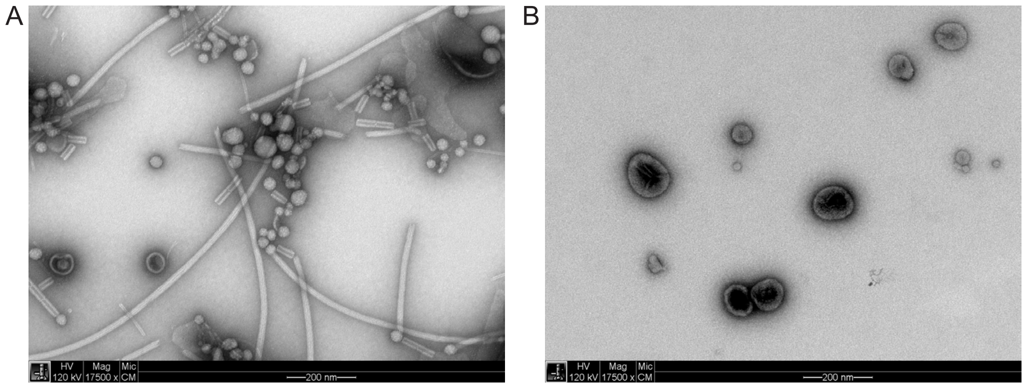

FIGURE S1. Density gradient ultracentrifugation efficiently purified bacterial outer membrane vesicles (OMV). Transmission electron microscope images showing OMV from *P. aeruginosa* prior to (A) and after (B) purification using a density gradient medium. Pili, flagellar fragments, and other bacterial components were removed by this procedure. The OMV were negatively stained and examined with CM120 Biotwin Philips electron microscope operating at 120 kV at 17,500× magnification. Micrographs were recorded with a Cantega G2 (Olympus SIS, Münster, Germany) 2k pixels charge-coupled device camera.

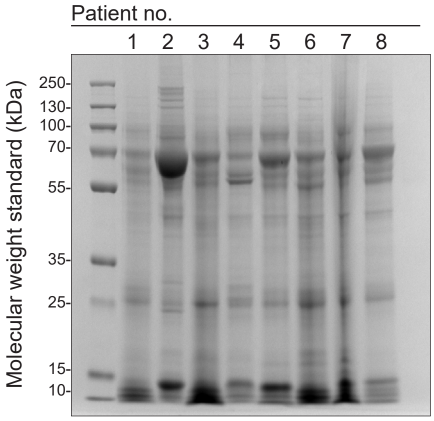

FIGURE S2. Protein profiles of included patient samples. BALF samples from pneumonia patients (numbers 1 through 8) were separated using SDS-PAGE and Coomassie-stained for sample quality control purposes.

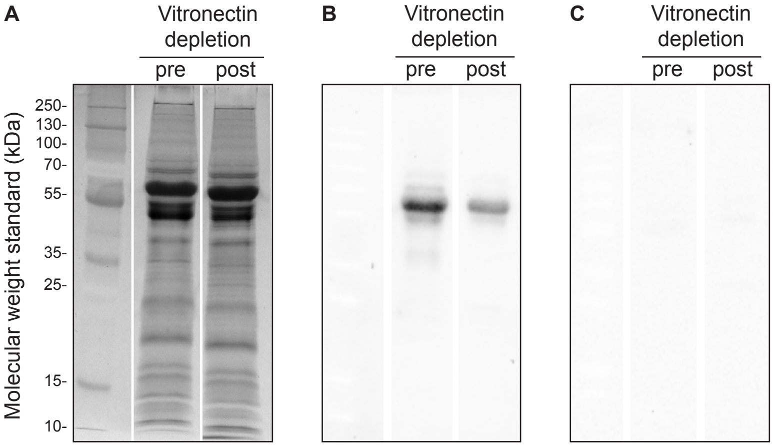

FIGURE S3. Vitronectin depletion of bronchoalveolar lavage fluid. BALF was depleted from vitronectin using anti-vitronectin antibodies coupled to sepharose protein A/G resin. No difference in protein composition was seen before (pre) and after (post) depletion (A). Immunoblotting revealed a distinct decrease in vitronectin concentration after the depletion (B). No unspecific background signal due was the primary antibody was omitted (C).

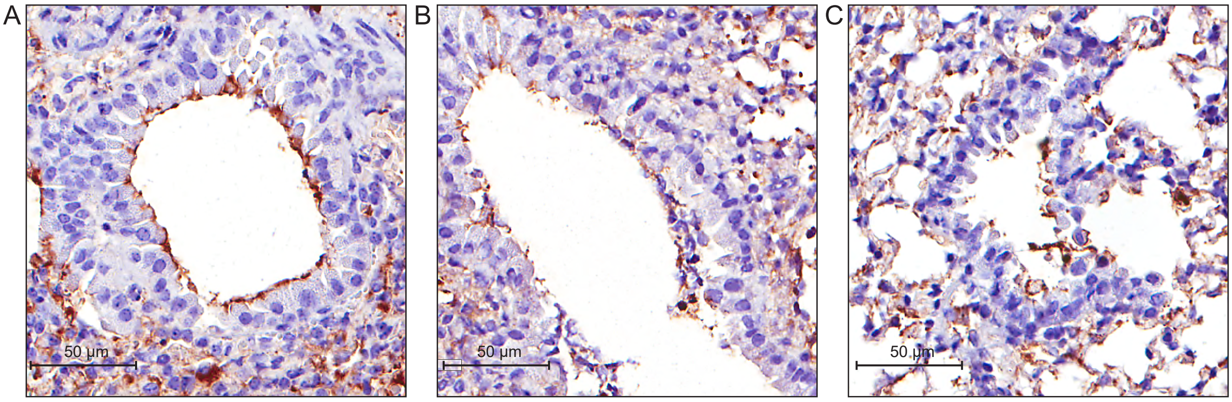

FIGURE S4. Vitronectin was detected in the mouse bronchoalveolar space and on the apical surface of bronchial epithelial cells 48 h after challenge with OMV, but no sign was found of intracellular vitronectin in bronchial epithelial cells. The mouse lung tissue was challenged with OMV from *H. influenzae* (A) or *P. aeruginosa* (B and C) and stained using immunohistochemistry for vitronectin (stained brown).

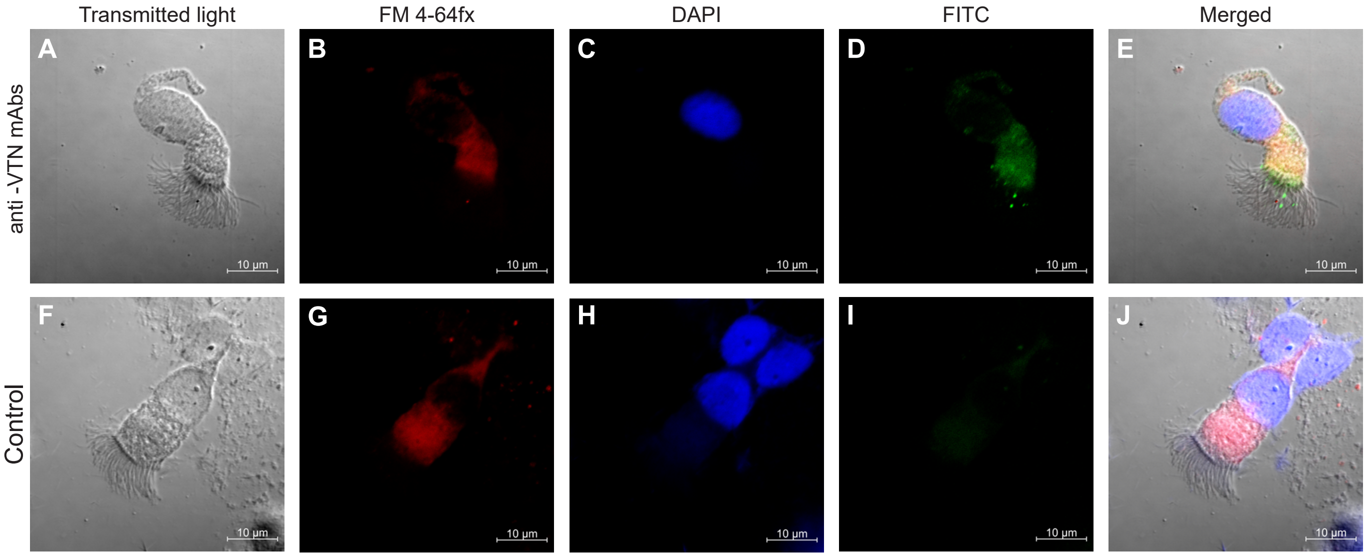

FIGURE S5. Vitronectin is present on the cell surface of bronchial epithelial cells from a pneumonia patient. Vitronectin was detected on the surface of ciliated bronchial epithelial

cells collected with protected brush from a patient with pneumonia. Upper panel, from left to right: (A) transmitted light image, (B) membranes and vacuoles counterstained red by FM 4-64fx (Invitrogen), (C) nuclei stained blue by DAPI (Dako), (D) vitronectin stained green by anti-vitronectin antibody VN58-1 and secondary FITC-conjugated antibodies, and (E) a merged picture. Lower panel: corresponding control micrograph where the anti-vitronectin antibody was omitted (F-J). In these experiments, cells were adhered to glass slides in triplicates by cytocentrifuging at 1000 rpm for 5 min, airdried and blocked with PBS with 20% tween and 2% BSA. Anti-vitronectin mAb (VN58-1; Abcam, Cambridge, UK) was added followed by washing, secondary FITC conjugated pAbs (Dako, Glostrup, Denmark) and repeated washing. Finally the slide was incubated with FM4-64fx 5 μg/mL in cold PBS for 1 min before addition of mounting media with 0.3 ug/ml DAPI (Dako). Images were acquired using a Zeiss LSM 700 confocal microscope with Plan-Apochromat 63x/1.40 and the accompanying Zen software (Zeiss, Oberkochen, Germany). Color channels were adjusted using the same parameters for the corresponding experiment and control pictures.
